# An ultra-small nine-color spectrometer with a two-layer biparted ten-dichroic-mirror array and an image sensor

**DOI:** 10.1038/s41598-022-20814-3

**Published:** 2022-10-03

**Authors:** Takashi Anazawa, Shuhei Yamamoto, Ryoji Inaba

**Affiliations:** 1 Research & Development Group, Hitachi Ltd., 1-280 Higashi-koigakubo, Kokubunji, Tokyo, 185-8601 Japan; 2 Analytical & Medical Solution Business Group, Hitachi High-Tech Corporation, 882 Ichige, Hitachinaka, Ibaraki 312-8504 Japan

**Keywords:** Analytical chemistry, Optical techniques, Characterization and analytical techniques, Imaging techniques, Optical spectroscopy

## Abstract

An ultra-small (54 × 58 × 8.5 mm) and large aperture (1 × 7 mm) nine-color spectrometer—using an array of ten dichroic mirrors “biparted” as two layers—was developed and used for snapshot spectral imaging. Incident-light flux with a cross section smaller than the aperture size is split into nine color fluxes with 20-nm-width contiguous wavelength bands and central wavelengths of 530, 550, 570, 590, 610, 630, 650, 670, and 690 nm. Images of the nine color fluxes are simultaneously and efficiently measured by an image sensor. Unlike a conventional dichroic-mirror array, the developed dichroic-mirror array has a unique biparted configuration that not only increases the number of colors that can be measured simultaneously but also improves the image resolution of each color flux. The developed nine-color spectrometer was used for four-capillary-array electrophoresis. Eight dyes concurrently migrating in each capillary were simultaneously quantified by nine-color laser-induced fluorescence detection. Since the nine-color spectrometer is not only ultra-small and inexpensive but also has high light throughput and sufficient spectral resolution for most spectral-imaging applications, it has the potential to be widely used in various fields.

## Introduction

Hyper- and multi-spectral imaging have become indispensable technologies in fields^1^ such as astronomy^2^, earth-observation remote sensing^3,4^, food and water quality control^5,6^, art conservation and archeology^7^, forensic medicine^8^, surgery^9^, and biomedical assay and diagnosis^10–13^. Methods for measuring the spectrum of the light emitted from each emission point in the field of view are categorized as (1) point scanning (“whisk-broom”)^14,15^, (2) line scanning (“push-broom”)^16–18^, (3) wavelength scanning^19–21^, and (4) snapshot^22–25^. In the case of all these methods, spatial resolution, spectral resolution, and temporal resolution share a trade-off relation^9,10,12,26^. In addition, light throughput has a significant effect on sensitivity, namely, signal-to-noise ratio in spectral imaging^26^. Light throughput, namely, light-utilization efficiency, is proportional to the ratio of the amount of light actually measured to the total amount of light in the wavelength band to be measured emitted from each emission point per unit time. When the intensity or spectrum of the light emitted from each emission point changes over time, or when the position of each emission point changes over time, category (4) is the appropriate method because the spectra of the lights emitted from all emission points are measured simultaneously^24^.

Most of the above-mentioned methods are combined with large, complicated, and/or expensive spectrometers using gratings^18^ or prisms^14,16,22,23^ for categories (1), (2), and (4) or filter wheels^20,21^, liquid–crystal tunable filters (LCTF)^25^, or acousto-optical tunable filters (AOTF)^19^ for category (3). In contrast, spectrometers using several dichroic mirrors for category (4) are small and inexpensive thanks to their simple configuration^27–30^. Moreover, they have high light throughput because both of the lights split by each dichroic mirror (that is, the transmitted light and the reflected light of the incident light on each dichroic mirror) are fully and continuously utilized. However, the number of wavelength bands (i.e., colors) to be measured simultaneously is limited to around four^26^.

Spectral imaging based on fluorescence detection is often used for multiplex analysis in biomedical assay and diagnosis^10,13^. In multiplex analysis, since multiple kinds of analytes (e.g., specific DNAs or proteins) are respectively labeled with different fluorescent dyes, the amount of each analyte present at each emission point in the field of view at each time is quantified by multicomponent analysis, i.e., unmixing the detected spectrum of fluorescences emitted from each emission point at each time^31,32^. During that procedure, different dyes, each of which emit different fluorescence, can co-localize, that is, coexist spatially and temporally. The current maximum number of dyes that can be excited by a single laser beam so that each dye is distinguishable from the others is eight^33^. This upper limit is not determined by spectral resolution (i.e., number of colors) but by the widths of the fluorescence spectra (≥ 50 nm) and the magnitudes of the Stokes shifts (≤ 200 nm) of the dyes when fluorescence resonance energy transfer (FRET)^10^ is used. However, the number of colors must be greater than or equal to the number of dyes in order to unmix the spectral overlaps of the dyes^31,32^. It is therefore necessary to increase the number of colors measured simultaneously to eight or more.

Recently, an ultra-small seven-color spectrometer (using a seven-dichroic-mirror array and an image sensor for measuring four fluorescence fluxes) was developed^31^. The spectrometer is two to three orders of magnitude smaller than a conventional spectrometer using a grating or a prism^34,35^. However, it is difficult to array more than seven dichroic mirrors in a spectrometer and simultaneously measure more than seven colors^36,37^. As the number of dichroic mirrors increases, the maximum difference in the optical-path lengths of the split color fluxes increases, and it becomes difficult to image all the fluxes on the same sensor plane. The longest optical-path length of the fluxes also increases, so the aperture width of the spectrometer (namely, the maximum width of the light analyzed by the spectrometer) decreases.

In response to the above-described issues, in this study, an ultra-small nine-color spectrometer—using a two-layer “biparted” ten-dichroic-mirror array and an image sensor—for snapshot spectral imaging [category (4)] was developed. The developed spectrometer has a smaller maximum optical-path-length difference and a shorter longest optical-path length than those of the previous spectrometer^31^. It was applied to four-capillary-array electrophoresis for detecting nine-color laser-induced fluorescence, and eight dyes concurrently migrating in each capillary were simultaneously quantified. Since the developed spectrometer is not only ultra-small and inexpensive but also has high light throughput and sufficient spectral resolution for most spectral-imaging applications, it can be widely used in various fields.

## Results

### Design of dichroic-mirror array

**Figure 1:**
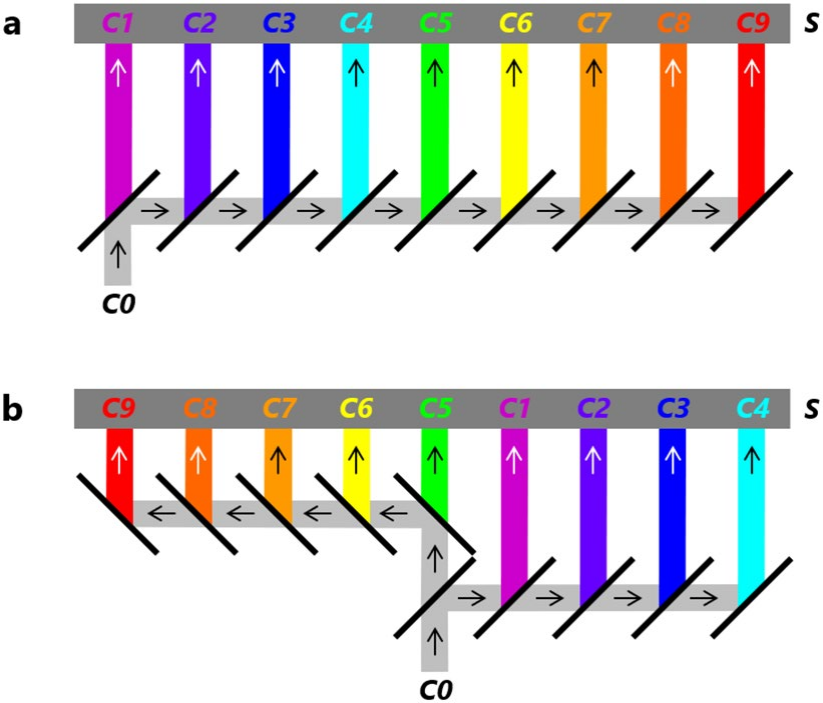
Schematic diagrams of conventional and novel nine-color spectrometers. (**a**) Conventional nine-color spectrometer with nine-dichroic-mirror array. (**b**) Novel nine-color spectrometer with two-layer biparted ten-dichroic-mirror array. Incident light flux ***C0*** is split into nine color light fluxes ***C1***−***C9*** and detected by image sensor ***S***.

A conventional nine-color spectrometer is illustrated in Fig. 1a. Its design follows the design of the previous ultra-small seven-color spectrometer^31^. It is composed of nine horizontally arrayed dichroic mirrors inclined at 45° to the right, and an image sensor (***S***) is located above the nine dichroic mirrors. An incoming flux (***C0***) from below is split by the nine-dichroic-mirror array into nine outgoing color fluxes (***C1***, ***C2***, ***C3***, ***C4***, ***C5***, ***C6***, ***C7***, ***C8***, and ***C9***) in the upward direction. All nine color fluxes are directly input into the image sensor and simultaneously detected. In this study, ***C1***, ***C2***, ***C3***, ***C4***, ***C5***, ***C6***, ***C7***, ***C8***, and ***C9*** are in wavelength order and shown in magenta, purple, blue, cyan, green, yellow, orange, red orange, and red, respectively. Although these color indications are used throughout this paper, as shown in Fig. 3, they differ from the colors actually observed by the human eye.

The developed novel nine-color spectrometer with a two-layer biparted ten-dichroic-mirror array and an image sensor is illustrated in Fig. 1b. On the lower layer, five dichroic mirrors are inclined at 45° to the right and arrayed from the center of the ten-dichroic-mirror array to the right. On the upper layer, the other five dichroic mirrors are inclined at 45° to the left and arrayed from the center to the left. The leftmost dichroic mirror on the lower layer and the rightmost dichroic mirror on the upper layer overlap each other. An incoming flux (***C0***) from below is split into four outgoing color fluxes (***C1***−***C4***) by the five dichroic mirrors on the right and five outgoing color fluxes (***C5***−***C9***) by the five dichroic mirrors on the left. In a similar manner to the conventional nine-color spectrometer, all nine color fluxes are directly input into the image sensor (***S***) and simultaneously detected. Comparing Fig. 1a and b shows that in the case of the novel nine-color spectrometer, both the maximum difference in the optical-path lengths and the longest optical-path length of the nine color fluxes are halved.

**Figure 2:**
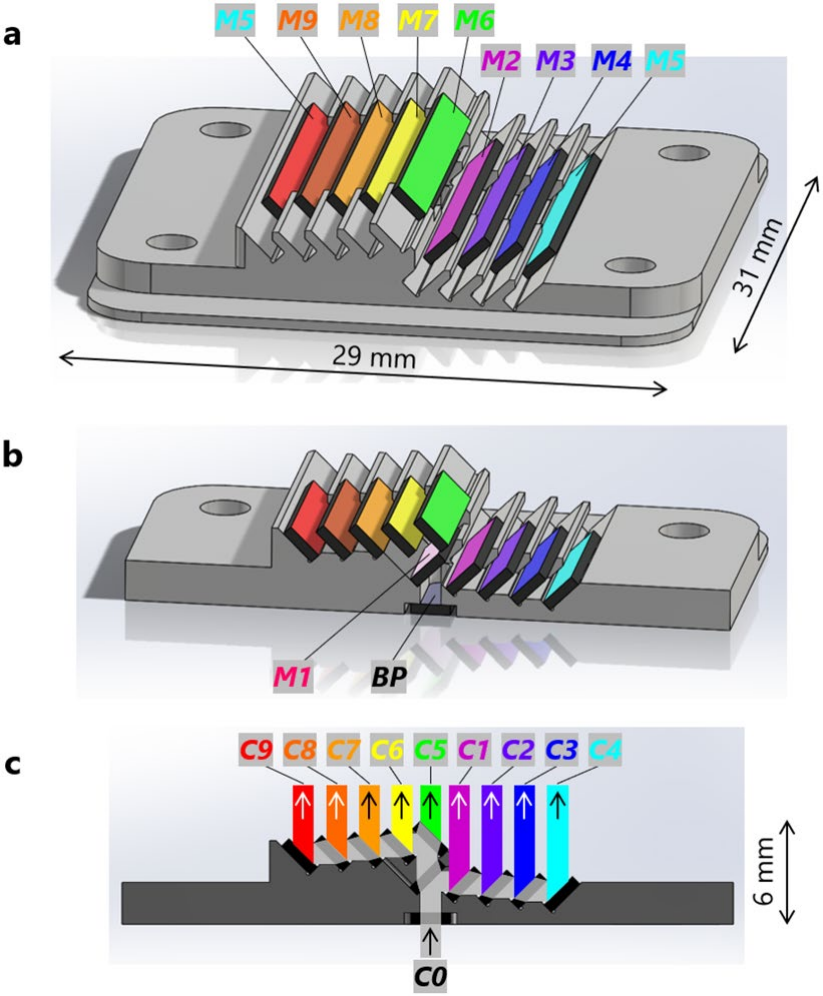
Design of two-layer biparted ten-dichroic-mirror array. (**a**) Perspective and (**b**) cross-sectional views of the two-layer biparted ten-dichroic-mirror array (with size of 29 mm × 31 mm × 6 mm). It consists of five dichroic mirrors (***M1***−***M5***) arrayed on the lower layer, five dichroic mirrors (***M6***−***M9*** and another ***M5***) arrayed on the upper layer, and a bandpass filter (***BP***) below ***M1***. (**c**) Cross-sectional view from the perpendicular direction, with ***C0*** and ***C1***−***C9*** overlaid.

The detailed design of the two-layer biparted ten-dichroic-mirror array—with an ultra-small size of 29 mm (width) × 31 mm (depth) × 6 mm (height)—is shown in Fig. 2. The ten-dichroic-mirror array is composed of five dichroic mirrors (***M1***−***M5***) on the right and five dichroic mirrors (***M6***−***M9*** and another ***M5***) on the left, each fixed on an aluminum holder. All the dichroic mirrors are arrayed in a stepwise manner to compensate parallel displacement of the fluxes due to refraction as the fluxes pass through the mirrors^31^. A bandpass filter (***BP***) is fixed below ***M1***. The size of ***M1*** and ***BP*** is 10 mm (long side) × 1.9 mm (short side) × 0.5 mm (thickness). That of the other dichroic mirrors is 15 mm × 1.9 mm × 0.5 mm. The array interval between ***M1*** and ***M2*** is 1.7 mm, and the array interval of the other dichroic mirrors is 1.6 mm. Incoming flux ***C0*** and the nine color fluxes ***C1***−***C9*** split by the ten-dichroic-mirror array are overlaid in Fig. 2c.

Aperture width in the horizontal direction, indicated by the width of ***C0*** in Fig. 2c, is 1 mm, and that in the direction perpendicular to the plane of Fig. 2c, specified by the design of the aluminum holder, is 7 mm. That is, the novel nine-color spectrometer has a large aperture size of 1 mm × 7 mm. Optical path length of ***C4*** is the longest among ***C1***−***C9***, and the optical path length of ***C4*** inside the ten-dichroic-mirror array specified by the above-mentioned ultra-small size (29 mm × 31 mm × 6 mm) is 12 mm. Meanwhile, optical path length of ***C5*** is the shortest among ***C1***−***C9***, and optical path length of ***C5*** is 5.7 mm. Maximum difference in the optical-path lengths is therefore 6.3 mm. The above optical path lengths are corrected for extension of optical lengths by light transmission through ***M1***−***M9*** and ***BP*** (made of quartz).

Spectral characteristics of ***M1***−***M9*** and ***BP*** were designed so that fluxes ***C1***, ***C2***, ***C3***, ***C4***, ***C5***, ***C6***, ***C7***, ***C8***, and ***C9*** have wavelength ranges of 520–540, 540–560, 560–580, 580–600, 600–620, 620–640, 640–660, 660–680, and 680–700 nm, respectively.

### Development of ultra-small nine-color spectrometer

**Figure 3:**
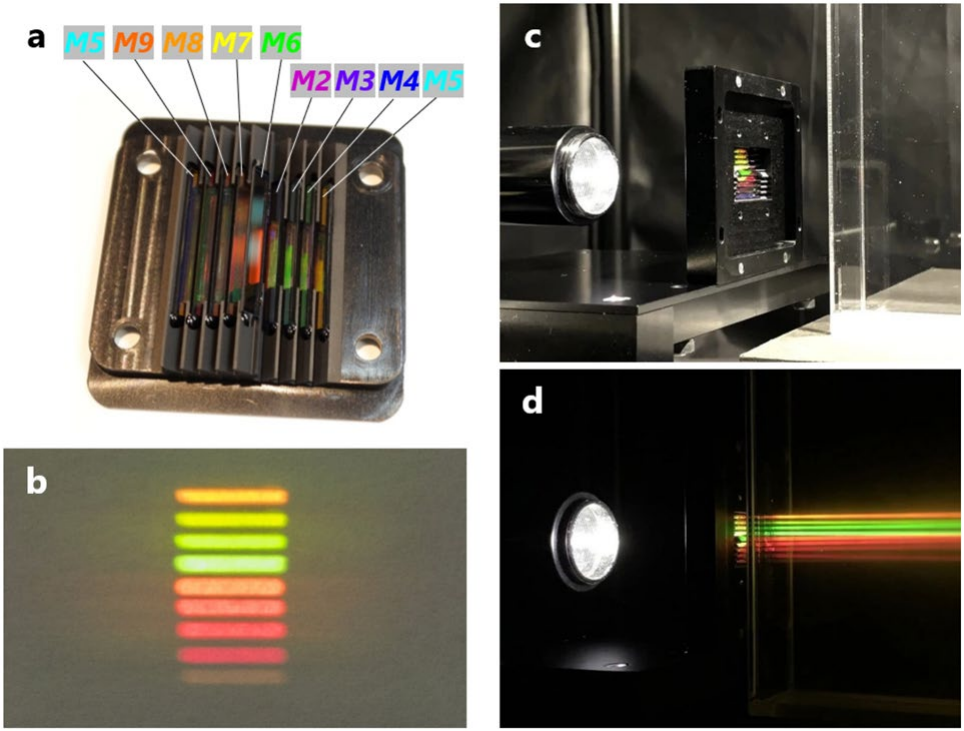
Fabricated ten-dichroic-mirror array and its demonstrations. (**a**) Fabricated ten-dichroic-mirror array. (**b**) Nine-color split images, with sizes of 1 mm × 7 mm, projected on a sheet of paper placed in front of the ten-dichroic-mirror array and illuminated by white light from behind. (**c**) Ten-dichroic-mirror array illuminated by white light from behind. (**d**) Nine-color split fluxes emitted from the ten-dichroic-mirror array, observed by placing a smoke-filled acrylic tank in front of the ten-dichroic-mirror array in **c** and darkening the room.

A photograph of the fabricated ten-dichroic-mirror array is shown in Fig. 3a. ***M1***−***M9*** and ***BP*** are respectively glued on 45° slopes and the horizontal plane of the aluminum holder, although ***M1*** and ***BP*** are hidden on the back side in the figure.

**Figure 4:**
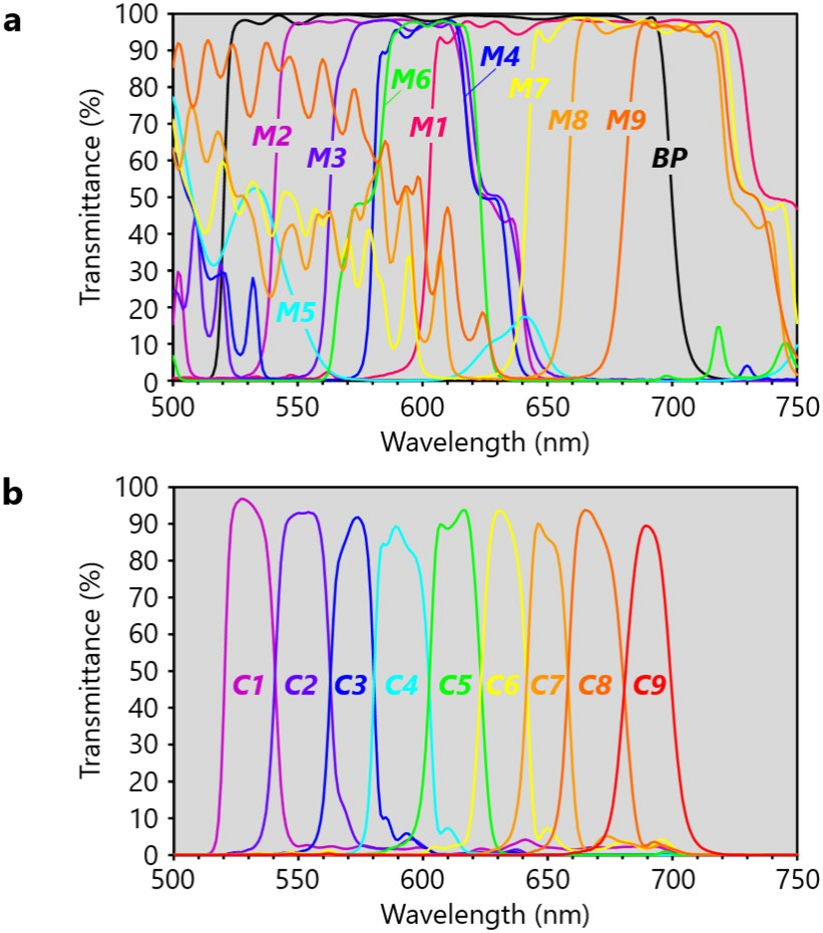
Transmission spectra of dichroic mirrors and split nine-color fluxes. (**a**) Measured transmission spectra of ***M1***−***M9*** at incident angles of 45° and ***BP*** at incident angle of 0°. (**b**) Transmission spectra of ***C1***–***C9*** relative to ***C0*** calculated from (**a**).

Measured transmission spectra of ***M1***−***M9*** for ***C0*** at incident angles of 45° and ***BP*** for ***C0*** at incident angle of 0° are shown in Fig. 4a. Transmission spectra of ***C1***−***C9*** relative to ***C0*** are shown in Fig. 4b. These spectra were calculated from the spectra in Fig. 4a on the basis of the optical paths of ***C1***–***C9*** in Figs. 1b and 2c. For example, TS(***C4***) = TS (***BP***) × [1 − TS (***M1***)] × TS (***M2***) × TS (***M3***) × TS (***M4***) × [1 − TS (***M5***)], and TS(***C9***) = TS (***BP***) × TS (***M1***) × [1 − TS (***M6***)] × TS (***M7***) × TS (***M8***) × TS (***M9***) × [1 − TS (***M5***)], where TS(***X***) and [1 − TS(***X***)] are respectively the transmission and reflection spectra of ***X***. As shown in Fig. 4b, transmission bands (≥ 50% transmittance) of ***C1***, ***C2***, ***C3***, ***C4***, ***C5***, ***C6***, ***C7***, ***C8***, and ***C9*** are 521–540, 541–562, 563–580, 581–602, 603–623, 624–641, 642–657, 659–680, and 682–699 nm, respectively. These results are consistent with the designed wavelength bands. Moreover, light-utilization efficiency of ***C0*** is high, that is, average maximum transmittance of ***C1***–***C9*** is 92%.

In Fig. 3c, the ten-dichroic-mirror array is positioned upright so that its right side in Fig. 3a is the upper side and a collimated LED white-light flux (***C0***) is illuminated from the back side. The ten-dichroic-mirror array shown in Fig. 3a is held inside an adapter with size of 54 mm (height) × 58 mm (depth) × 8.5 mm (thickness). In Fig. 3d, in addition to the state shown in Fig. 3c, a smoke-filled acrylic tank is placed in front of the ten-dichroic-mirror array, and the room light is turned off. As a result, nine color split fluxes emitted from the ten-dichroic-mirror array are visible in the tank. Each split flux has a rectangular cross section with size of 1 × 7 mm, which corresponds to the aperture size of the novel nine-color spectrometer. In Fig. 3b, a piece of paper is placed in front of the ten-dichroic-mirror array in Fig. 3c and 1 x 7-mm images of the nine color split fluxes projected on the paper are observed from the traveling direction of the nine color split fluxes. The nine color split fluxes in Fig. 3b and d are ***C4***, ***C3***, ***C2***, ***C1***, ***C5***, ***C6***, ***C7***, ***C8***, and ***C9*** in order from top to bottom, as also understood from Figs. 1b and 2c. These are observed in colors corresponding to their respective wavelengths. Since intensity of the LED white light (see Supplementary Fig. S3) and sensitivity of the color camera used for taking photographs in Fig. 3 in regard to ***C9*** (682–699 nm) are both low, the observed intensity of ***C9*** (the flux at the bottom) is weaker than that of other split fluxes. Similarly, ***C9*** was faintly observed with the naked eye. Meanwhile, ***C2*** (the second flux from the top) looks green in Fig. 3, but looks more yellow to the naked eye.

The progress from Fig. 3c to d is shown in Supplementary Video 1. Immediately after the LED white-light flux is transmitted through the ten-dichroic-mirror array, it is simultaneously split into the nine color fluxes. Eventually, the smoke in the tank disappears gradually from the top to the bottom of the tank, so the nine color split fluxes also disappear in order from the top to the bottom. On the contrary, in Supplementary Video 2, when the wavelength of the light flux incident on the ten-dichroic-mirror array is sequentially changed from long to short in the order 690, 671, 650, 632, 610, 589, 568, 550, and 532 nm, only the corresponding color split flux among the nine color split fluxes is visualized in the order ***C9***, ***C8***, ***C7***, ***C6***, ***C5***, ***C4***, ***C3***, ***C2***, and ***C1***. The acrylic tank was replaced with a quartz cell so that it was possible to clearly observe the sheet shape of each split flux from diagonally above. In addition, Supplementary Video 3 is edited so that the wavelength-change portion in Supplementary Video 2 is repeatedly reproduced. It is the most eloquent expression of the features of the ten-dichroic-mirror array.

The above-described results show that the fabricated ten-dichroic-mirror array, or the novel nine-color spectrometer, functions as designed. The novel nine-color spectrometer was formed by mounting the ten-dichroic-mirror array with the adapter directly on an image-sensor board.

### Spectroscopic analysis of lights emitted from four-emission points

**Figure 5:**
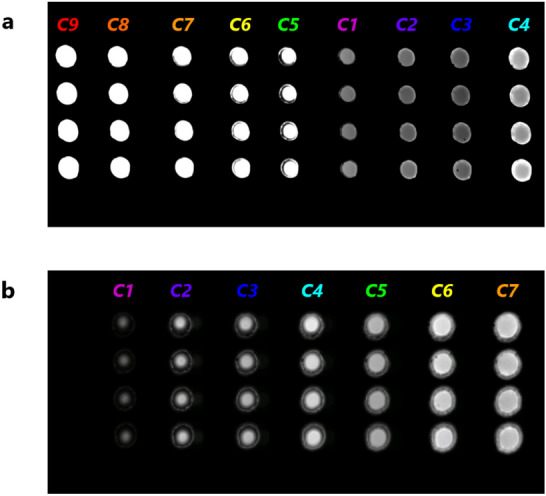
Comparison of qualities of split images formed by the conventional and novel spectrometers. (**a**) Four sets of nine-color split images (***C1***−***C9***) formed by the novel nine-color spectrometer. (**b**) Four sets of seven-color split images (***C1***−***C7***) formed by the conventional seven-color spectrometer. Fluxes (***C0***) with wavelength of 400 to 750 nm from four emission points were respectively collimated and incident on each spectrometer.

Light fluxes with a wavelength band of 400 to 750 nm, emitted from four ϕ50-μm emission points arranged at 1-mm intervals in the direction perpendicular to the plane of Fig. 2c, were respectively collimated by the four-lens array used in the previous study^31,34^. The four-lens array consists of four ϕ1-mm lenses with focal lengths of 1.4 mm and intervals of 1 mm. The four collimated fluxes (four ***C0***s) arranged at 1-mm intervals were incident on ***BP*** of the novel nine-color spectrometer. Each flux (***C0***) was split into nine color fluxes (***C1***−***C9***) by the ten-dichroic-mirror array. The resulting thirty-six fluxes (four sets of ***C1***−***C9***) were then directly input into a CMOS image sensor (***S***) directly attached to the ten-dichroic-mirror array. As a result, as shown in Fig. 5a, all the images of the thirty-six fluxes were simultaneously and clearly detected in uniform size because of both the small maximum-optical-path-length difference and the short maximum optical-path length. Depending on the spectrum of the incident fluxes (see Supplementary Fig. S4), image intensities of the four sets of ***C1***, ***C2***, and ***C3*** are relatively low. The size of the thirty-six images is 0.57 ± 0.05 mm (average ± standard deviation). The image magnification is therefore 11.4 on average. The vertical image interval is 1 mm (the same as the lens-array interval), and the horizontal image interval is 1.6 mm (the same as the dichroic-mirror-array interval) on average. Since the image size is thus sufficiently smaller than the image intervals, each image can be measured independently (with low crosstalk). Meanwhile, images of twenty-eight fluxes detected by the conventional seven-color spectrometer used in our previous study^31^ are shown in Fig. 5b. The seven-dichroic-mirror array was created by removing the two dichroic mirrors on the far right from the nine-dichroic-mirror array in Fig. 1a. Not all the images are in focus, and the size of the images increases from ***C1*** to ***C7***. The size of the twenty-eight images is 0.70 ± 0.19 mm. It is therefore difficult to maintain high imaging resolution in all the images. Coefficient of variation (CV) of the size of the twenty-eight images in Fig. 5b is 28%, whereas that of the size of the thirty-six images in Fig. 5a is reduced to 9%. The above-described results clarify that the novel nine-color spectrometer not only increased the number of colors measured simultaneously from seven to nine but also obtained high imaging resolution for each color.

**Figure 6:**
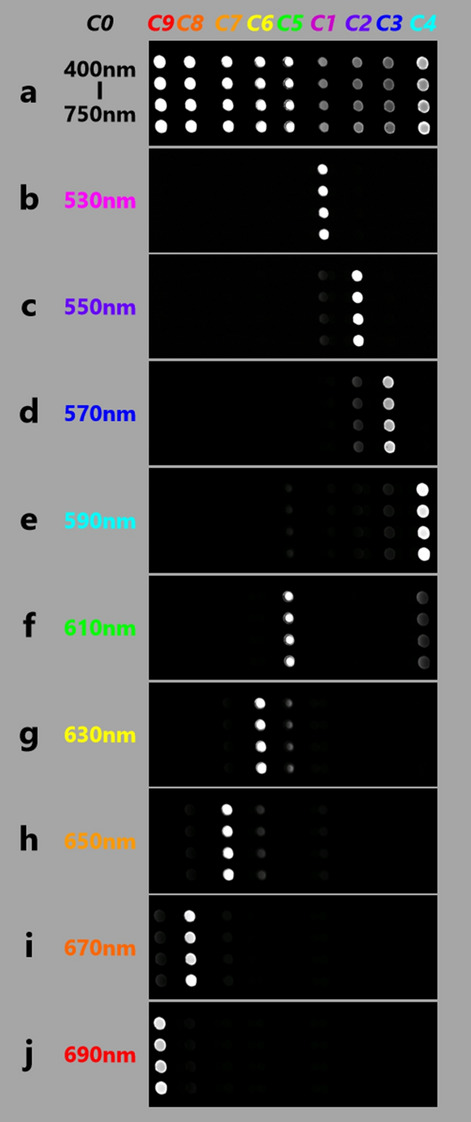
Spectroscopic performance of nine-color spectrometer. Four sets of nine-color split images (***C1***−***C9***) formed by the novel nine-color spectrometer when incident lights (four ***C0***s) are lights with wavelengths of (**a**) 400−750 nm (as in Fig. 5a), (**b**) 530 nm, (**c**) 550 nm, (**d**) 570 nm, (**e**) 590 nm, (**f**) 610 nm, (**g**) 630 nm, (**h**) 650 nm, (**i**) 670 nm, and (**j**) 690 nm, respectively.

Spectroscopic performance of the nine-color spectrometer was experimentally evaluated, and the evaluation results are shown in Fig. 6. Note that Fig. 6a shows the same result as Fig. 5a; that is, when each of the four ***C0***s had a wavelength of 400 to 750 nm, all the thirty-six images (the four sets of ***C1*** − ***C9***) were detected. On the contrary, as shown in Fig. 6b−j, when each ***C0*** had a specific wavelength of 530, 550, 570, 590, 610, 630, 650, 670, or 690 nm, almost only four corresponding images (four sets of ***C1***, ***C2***, ***C3***, ***C4***, ***C5***, ***C6***, ***C7***, ***C8***, or ***C9***) were detected. However, some images adjacent to the four corresponding images were very faintly detected, because the transmission spectra of ***C1***−***C9*** shown in Fig. 4b slightly overlap, and each ***C0*** of the specific wavelength has a wavelength band of 10 nm as described in Methods. These results are consistent with the transmission spectra of ***C1***−***C9*** shown in Fig. 4b and Supplementary Videos 2 and 3. In other words, the nine-color spectrometer functions as expected from the results shown in Fig. 4b. It is therefore concluded that the image-intensity profile of ***C1***−***C9*** represents the light spectrum of each ***C0***.

### Simultaneous quantification of eight dyes for four-capillary-array electrophoresis

**Figure 7:**
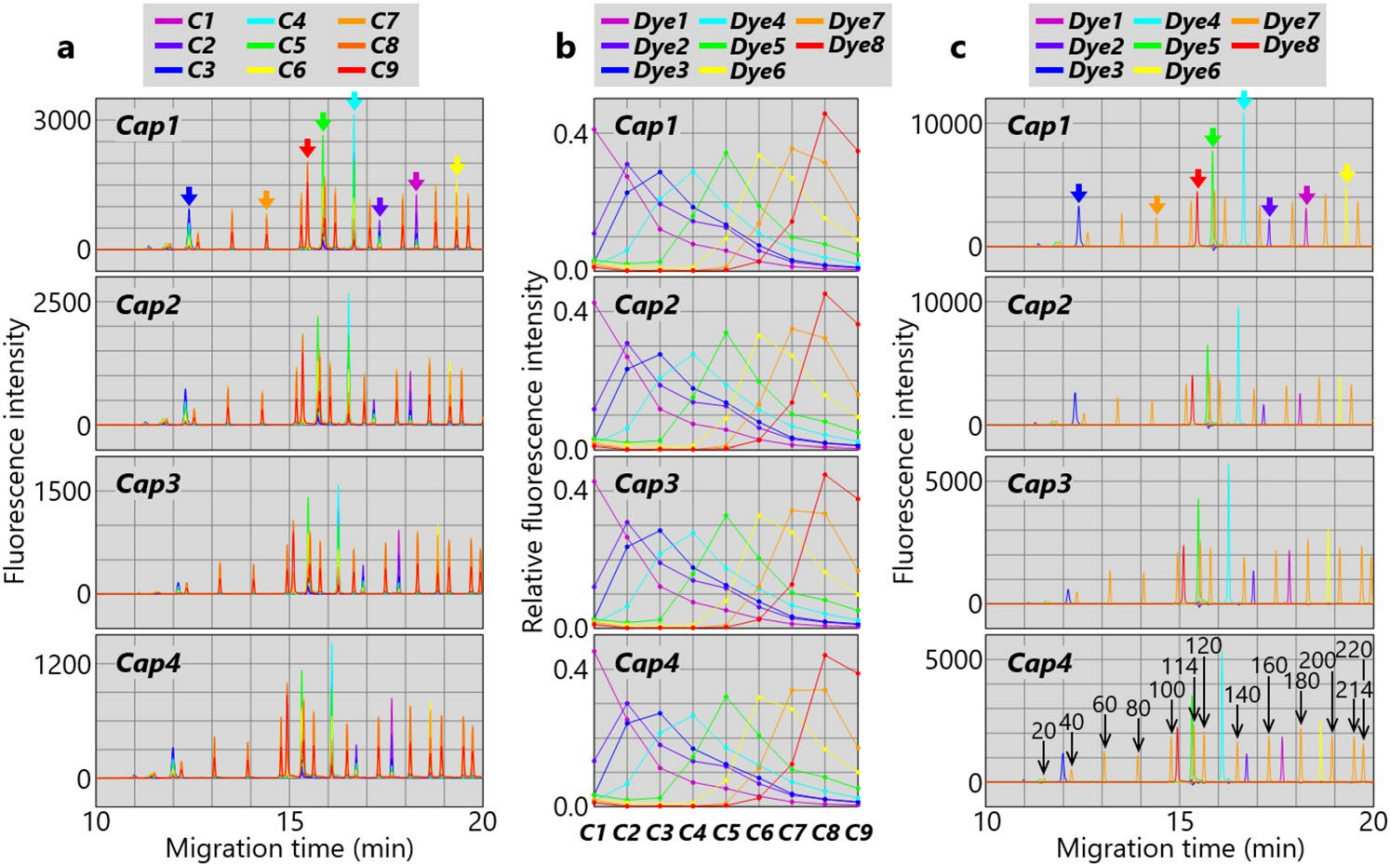
Simultaneous eight-dye quantification by nine-color spectrometer used for four-capillary-array electrophoresis. (**a**) Nine-color (***C1***−***C9***) electropherogram for each capillary. Eight peaks indicated by arrows for ***Cap1*** show individual fluorescence emission of eight dyes (***Dye1***−***Dye8***). Colors of the arrows correspond to those in (**b**) and (**c**). (**b**) Fluorescence spectra of eight dyes (***Dye1***−***Dye8***) for each capillary. **c** Eight-dye (***Dye1***−***Dye8***) electropherogram for each capillary. Peaks of ***Dye7***-labeled DNA fragments are indicated by arrows with their base lengths for ***Cap4***.

The developed nine-color spectrometer was applied to four-capillary-array electrophoresis (refer to Supplementary Material for details)^31,34,35^. The four-capillary array consists of four capillaries (with outer diameters of 360 μm and inner diameters of 50 μm) arranged at 1-mm intervals at laser-irradiation positions. A sample containing DNA fragments labeled with eight kinds of dyes, namely, FL-6C (***Dye1***), JOE-6C (***Dye2***), dR6G (***Dye3***), TMR-6C (***Dye4***), CXR-6C (***Dye5***), TOM-6C (***Dye6***), LIZ (***Dye7***), and WEN (***Dye8***) in ascending order of fluorescence-emission wavelength, was separated in each of the four capillaries (hereafter, ***Cap1***, ***Cap2***, ***Cap3***, and ***Cap4***). Laser-induced fluorescences from ***Cap1***−***Cap4*** were collimated by the four-lens array and simultaneously detected by the nine-color spectrometer. Time courses of nine-color (***C1***−***C9***) fluorescence intensities during electrophoresis, i.e., a nine-color electropherogram for each capillary, are shown in Fig. 7a. Equivalent nine-color electropherograms were obtained in ***Cap1***−***Cap4***. As indicated by the arrows in Fig. 7a for ***Cap1***, eight peaks in each nine-color electropherogram respectively show individual fluorescence emissions of ***Dye1***−***Dye8***.

Intensity distributions of ***C1***−***C9*** at the eight peaks are respectively shown in Fig. 7b. Since ***C1***−***C9*** and ***Dye1***−***Dye8*** are both in wavelength order, the eight distributions in Fig. 7b show fluorescence spectra of ***Dye1***−***Dye8*** in order from left to right. In this study, ***Dye1***, ***Dye2***, ***Dye3***, ***Dye4***, ***Dye5***, ***Dye6***, ***Dye7***, and ***Dye8*** are shown in magenta, purple, blue, cyan, green, yellow, orange, and red, respectively. Note that the colors of the arrows in Fig. 7a correspond to the colors of the dyes in Fig. 7b. Fluorescence intensities of ***C1***−***C9*** for each spectrum in Fig. 7b are normalized so that their summation is one. Eight equivalent fluorescence spectra are obtained by ***Cap1***−***Cap4***. Fluorescence spectral overlaps between ***Dye1***−***Dye8*** are clearly observed.

As shown in Fig. 7c, for each capillary, the nine-color electropherogram in Fig. 7a was converted to an eight-dye electropherogram by multicomponent analysis^31^ based on the eight fluorescence spectra in Fig. 7b (refer to Supplementary Material for details). Since the fluorescence spectral overlaps in Fig. 7a do not appear in Fig. 7c, it is possible to individually identify and quantify ***Dye1***−***Dye8*** at each time point even if different quantities of ***Dye1***−***Dye8*** emit fluorescences simultaneously. That would not be possible by conventional seven-color detection^31^; however, it is possible by the developed nine-color detection. As indicated by arrows in Fig. 7c for ***Cap1***, only single peaks of fluorescence emissions of ***Dye3*** (blue), ***Dye8*** (red), ***Dye5*** (green), ***Dye4*** (cyan), ***Dye2*** (purple), ***Dye1*** (magenta), and ***Dye6*** (yellow) are observed in chronological order as expected. As for fluorescence emission of ***Dye7*** (orange), in addition to a single peak indicated by the orange arrow, several other single peaks are observed. That result is due to the fact that the sample contains Size Standards, namely, ***Dye7***-labeled DNA fragments with various base lengths. As indicated in Fig. 7c for ***Cap4***, those base lengths include 20, 40, 60, 80, 100, 114, 120, 140, 160, 180, 200, 214, and 220 base lengths.

## Discussion

The key features of the developed nine-color spectrometer using the two-layer biparted ten-dichroic-mirror array are its small size and simple configuration. Since the ten-dichroic-mirror array held inside the adapter shown in Fig. 3c is directly mounted on the image-sensor board (refer to Figs. S1 and S2), the size of the nine-color spectrometer is the same as the size of the adapter, that is, 54 × 58 × 8.5 mm (thickness). This ultra-small size is two to three orders of magnitude smaller than the size of a conventional spectrometer using a grating or a prism. Moreover, since the nine-color spectrometer is configured so that light is incident perpendicularly to the image-sensor surface, it is easy to allocate space for the nine-color spectrometer in a system, such as a microscope, a flow cytometer, or a capillary-array electrophoresis analyzer, in a manner that further miniaturizes the system. Meanwhile, the sizes of the ten dichroic mirrors and the bandpass filter used in the nine-color spectrometer are only 10 × 1.9 × 0.5 mm or 15 × 1.9 × 0.5 mm. Therefore, more than 100 such small dichroic mirrors and bandpass filters can be respectively cut from 60-mm-square dichroic mirrors and bandpass filters. Consequently, it is possible to manufacture the ten-dichroic-mirror array at low cost.

Another feature of the nine-color spectrometer is its excellent spectroscopic performance. In particular, it enables, snapshot spectral imaging, i.e., simultaneous acquisition of images with spectral information. A continuous spectrum in the wavelength range from 520 to 700 nm with a resolution of 20 nm is obtained for each image. In other words, for each image, light intensities of the nine colors, i.e., the nine 20-nm wavelength bands that evenly divide the wavelength range from 520 to 700 nm, are detected. It is possible to adjust the wavelength range and each width of the nine wavelength bands by changing the spectral characteristics of the dichroic mirrors and the bandpass filter. Nine-color detection is useful not only in fluorescence measurement by spectral imaging (as described in this report) but also in many other general applications using spectral imaging. Although hundreds of colors can be detected with hyperspectral imaging, it has been found that multiple targets in the field of view can be identified with sufficiently high accuracy in many applications even if the number of colors to be detected is significantly reduced^38–40^. Since spatial resolution, spectral resolution, and temporal resolution share a trade-off relation in spectral imaging, reducing the number of colors can improve spatial resolution and temporal resolution. It also makes it possible to use a simple spectrometer like the one developed in this study and further reduce the calculation load.

In this study, eight dyes were simultaneously quantified by spectral unmixing of overlapping fluorescence spectra of the eight dyes based on nine-color detection. Up-to-nine kinds of dyes that coexist temporally and spatially can also be simultaneously quantified. The particular advantages of the nine-color spectrometer are its high light throughput and large aperture (1 × 7 mm). The ten-dichroic-mirror array has maximum transmittance of 92% of the light incident from the aperture in each of the nine wavelength bands. Utilization efficiency of the incident light in the wavelength range of 520 to 700 nm is almost 100%. In such a wide wavelength range, such high utilization efficiency cannot be obtained with any kind of diffraction grating. Even if the diffraction grating has diffraction efficiency of more than 90% at a specific wavelength, the diffraction efficiency at another wavelength decreases as the difference between that wavelength and the specific wavelength increases^41^. The aperture width in the direction perpendicular to the plane of Fig. 2c can be expanded from 7 mm to the width of an image sensor, e.g., 13 mm in the case of the image sensor used in this study, by slightly changing the design of the ten-dichroic-mirror array.

The nine-color spectrometer can be applied not only to capillary-array electrophoresis, as shown in this study, but also to various other applications. For example, as shown below, the nine-color spectrometer can be applied to a fluorescence microscope. A sample plane is imaged on the image sensor of the nine-color spectrometer by a 10 × objective lens. The optical distance between the objective lens and the image sensor is 200 mm, while the optical distance between the incident surface of the nine-color spectrometer and the image sensor is only 12 mm. Therefore, the image is approximately cut out at the incident surface to the aperture size (1 × 7 mm) and is divided into nine color images. That is, it is possible to perform nine-color snapshot spectral imaging of a region with size of 0.1 × 0.7 mm on the sample plane. In addition, by scanning the sample with respect to the objective lens in the horizontal direction of Fig. 2c, nine-color spectral imaging of a larger area on the sample plane is possible.

## Methods

### Components of ten-dichroic-mirror array

Components of the ten-dichroic-mirror array, i.e., ***M1***−***M9*** and ***BP***, were custom-made by Asahi Spectra Co., Ltd. using standard deposition techniques. Multiple thin layers of the dielectric material were respectively deposited on ten 60 × 60-mm quartz plates with thickness of 0.5 mm to meet the following specifications: ***M1***: IA = 45°, R ≥ 90% at 520–590 nm, T_ave_ ≥ 90% at 610–700 nm; ***M2***: IA = 45°, R ≥ 90% at 520–530 nm, T_ave_ ≥ 90% at 550–600 nm; ***M3***: IA = 45°, R ≥ 90% at 540–550 nm, T_ave_ ≥ 90% at 570–600 nm; ***M4***: IA = 45°, R ≥ 90% at 560–570 nm, T_ave_ ≥ 90% at 590–600 nm; ***M5***: IA = 45°, R ≥ 98% at 580–600 nm, R ≥ 98% at 680–700 nm; ***M6***: IA = 45°, T_ave_ ≥ 90% at 600–610 nm, R ≥ 90% at 630–700 nm; ***M7***: IA = 45°, R ≥ 90% at 620–630 nm, T_ave_ ≥ 90% at 650–700 nm; ***M8***: IA = 45°, R ≥ 90% at 640–650 nm, T_ave_ ≥ 90% at 670–700 nm; ***M9***: IA = 45°, R ≥ 90% at 650–670 nm, T_ave_ ≥ 90% at 690–700 nm; ***BP***: IA = 0°, T ≤ 0.01% at 505 nm, T_ave_ ≥ 95% at 530–690 nm, T ≥ 90% at 530–690 nm, T ≤ 1% at 725–750 nm, where IA, T, T_ave_, and R are incident angle, transmittance, averaged transmittance, and reflectance of unpolarized light.

### Demonstration of ten-dichroic-mirror array

White light (***C0***) with a wavelength range of 400–750 nm emitted from an LED light source (AS 3000, AS ONE CORPORATION) was collimated and vertically incident on ***BP*** of the ten-dichroic-mirror array. The spectrum of the LED white light is shown in Supplementary Fig. S3. An acrylic tank (with a size of 150 × 150 × 30 mm) was placed just in front of the ten-dichroic-mirror array, opposite ***BP***. Then, smoke made by submerging dry ice in water was poured into the acrylic tank to visualize the nine-color split fluxes of ***C1***–***C9*** emitted from the ten-dichroic-mirror array.

Alternatively, the collimated white-light flux (***C0***) was passed through one filter before being incident on ***BP***. The filter was initially an ND filter with OD of 0.6. It was then replaced with nine 10-nm bandpass filters with central wavelengths of 690, 671, 650, 632, 610, 589, 568, 550, and 532 nm in sequence at 5-s intervals by using a motorized filter wheel (FW212C, Thorlabs, Inc.). Finally, the ND filter was put back. The transmission bands of the nine bandpass filters correspond to those of ***C9***, ***C8***, ***C7***, ***C6***, ***C5***, ***C4***, ***C3***, ***C2***, and ***C1***, respectively. A quartz cell with inner size of 40 (optical length) × 42.5 (height) × 10 mm (width) was placed just in front of the ten-dichroic-mirror array, opposite ***BP***. Smoke was then supplied to the quartz cell through a tube so that the smoke concentration in the quartz cell was maintained to visualize the nine-color split fluxes of ***C1***–***C9*** emitted from the ten-dichroic-mirror array.

Videos of the nine-color split fluxes being emitted from the ten-dichroic-mirror array were shot in time-lapse mode on an iPhone XS. Images of the scene were captured at 1 frame per second, and the images were compiled to create the videos at 30 frames per second (for Supplementary Video 1) or 24 frames per second (for Supplementary Videos 2 and 3).

### Creation of four-emission points

A 50-μm-thick stainless-steel sheet (with four ϕ50-μm pinholes at 1-mm intervals) was placed on a diffuser plate. Light with a wavelength band of 400–750 nm, obtained by passing a halogen-lamp light through a shortpass filter with cut-off wavelength of 700 nm, was irradiated onto the diffuser plate. The spectrum of the light is shown in Supplementary Fig. S4. Alternatively, the light was also passed through one of 10-nm bandpass filters with central wavelengths of 530, 550, 570, 590, 610, 630, 650, 670, and 690 nm and irradiated onto the diffuser plate. As a result, four ϕ50-μm emission points with various wavelengths were formed on the stainless-steel sheet opposite the diffuser plate.

### Nine-color spectrometer for four-capillary array

The four-capillary array with the four-lens array was mounted on the nine-color spectrometer as shown in Figs. S1 and S2. The four-capillary array and the four-lens array are the same as those used in the previous study^31,34^. A 505-nm laser beam with power of 15 mW was simultaneously and uniformly irradiated onto the emission points of the four capillaries from the side direction. Fluorescence emitted from each of the emission points was collimated by the corresponding lens and split into nine color fluxes by the ten-dichroic-mirror array. The resulting thirty-six fluxes were then directly input into a CMOS image sensor (C11440–52U, Hamamatsu Photonics K·K.), and their images were simultaneously detected.

### Sample preparation and capillary electrophoresis

For each capillary, a 20-μl sample was prepared by mixing 1 μl of PowerPlex® 6C Matrix Standard (Promega Corporation), 1 μl of A Mix in ABI PRISM® BigDye® Primer Cycle Sequencing Ready Reaction Kit (Applied Biosystems), 4 μl of GeneScan™ 600 LIZ™ dye Size Standard v2.0 (Thermo Fisher Scientific), and 14 μl of water. The PowerPlex® 6C Matrix Standard includes six DNA fragments respectively labeled with the six dyes FL-6C, JOE-6C, TMR-6C, CXR-6C, TOM-6C, and WEN in the order of maximum emission wavelength. The base lengths of these DNA fragments are not disclosed, but it is known that WEN, CXR-6C, TMR-6C, JOE-6C, FL-6C, and TOM-6C labeled DNA fragments are in the order of base length. The A Mix in ABI PRISM® BigDye® Primer Cycle Sequencing Ready Reaction Kit includes a DNA fragment labeled with the dye dR6G. The base length of the DNA fragment is also not disclosed. The GeneScan™ 600 LIZ™ dye Size Standard v2.0 includes thirty-six DNA fragments labeled with the dye LIZ. The base lengths of these DNA fragments are 20, 40, 60, 80, 100, 114, 120, 140, 160, 180, 200, 214, 220, 240, 250, 260, 280, 300, 314, 320, 340, 360, 380, 400, 414, 420, 440, 460, 480, 500, 514, 520, 540, 560, 580, and 600 bases. The sample was denatured at 94 °C for 3 min and cooled on ice for 5 min. The sample was injected into each capillary at 26 V/cm for 9 s, and separated in each capillary filled with a POP-7™ polymer solution (Thermo Fisher Scientific), with effective length of 36 cm, and at 181 V/cm and 60 °C.

## Supplementary Information

Supplementary Information.

Supplementary Video 1.

Supplementary Video 2.

Supplementary Video 3.

## Data Availability

All data generated or analyzed during this study are included in this published article and its supplementary information. Additional data related to this study are available from the corresponding author on reasonable request.
