# Supplementary material for: An ultra-small nine-color spectrometer with a two-layer biparted ten-dichroic-mirror array and an image sensor
Source: Sci Rep. 2022 Oct 3;12:16518. doi: 10.1038/s41598-022-20814-3 (PMC9529936; doi:10.1038/s41598-022-20814-3)
Supplement: Supplementary file 1 — Supplementary Information. [file 41598_2022_20814_MOESM1_ESM.pdf]

Supplementary Material for

An ultra-small nine-color spectrometer with a two-layer biparted ten-dichroic-mirror array and an image sensor

Takashi Anazawa[1*], Shuhei Yamamoto[2], and Ryoji Inaba[2]

[1] Research & Development Group, Hitachi Ltd., 1-280 Higashi-koigakubo, Kokubunji, Tokyo, 185-8601, Japan
[2] Analytical & Medical Solution Business Group, Hitachi High-Tech Corporation, 882 Ichige, Hitachinaka, Ibaraki, 312-8504, Japan

[*]Corresponding author
Email: takashi.anazawa.rc@hitachi.com

Nine-color spectrometer for four-capillary array

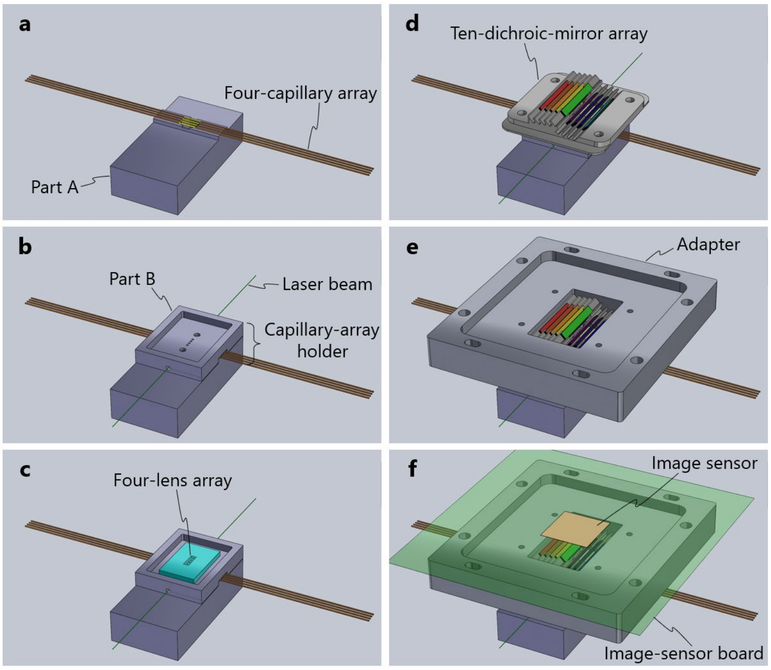

Fig. S1: Assembly of nine-color spectrometer for four-capillary array

a–**b** Peripheral parts of detection windows of four capillaries are arrayed at 1-mm intervals on the same plane to form a four-capillary array. This is achieved by sandwiching the peripheral parts between parts A and B of a capillary-array holder. The detection windows are simultaneously irradiated by introducing a laser beam from the side of the plane to form four-emission points on the detection windows. **c** A four-lens array is connected to the capillary-array holder to collimate light emitted from each emission point. **d**–**e** A ten-dichroic-mirror array held inside an adapter is connected to the capillary-array holder in **c** to split the collimated flux emitted from each emission point into nine color fluxes. **f** An image-sensor board is connected to the adapter in **e** to simultaneously detect the nine color fluxes from each emission point by an image sensor on the image-sensor board.

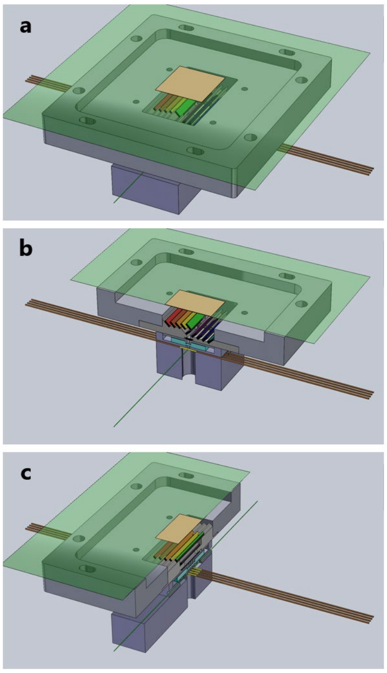

Fig. S2: Configuration of nine-color spectrometer for four-capillary array

a The nine-color spectrometer for the four-capillary array also shown in Fig. S1f. **b** Cross section of the nine-color spectrometer parallel to each capillary and perpendicular to the image sensor. **c** Cross section of the nine-color spectrometer parallel to the laser beam and perpendicular to the image sensor. For clarity, the laser beam and the capillaries are not cross-sectioned in each cross-section.

Multicomponent analysis

For ***Capj***, (***j*** = ***1***, ***2***, ***3***, and ***4***), fluorescence spectra of ***Dye1***−***Dye8*** shown in Fig. 7b are represented by the following 9 × 8 matrixes ***Aj***, respectively.

$$\boldsymbol{A_1} = \begin{pmatrix} 0.411 & 0.110 & 0.027 & 0.016 & 0.032 & 0.021 & 0.020 & 0.012 \\ 0.274 & 0.310 & 0.227 & 0.060 & 0.022 & 0.014 & 0.003 & 0.002 \\ 0.122 & 0.195 & 0.287 & 0.210 & 0.028 & 0.010 & 0.002 & 0.003 \\ 0.079 & 0.145 & 0.186 & 0.288 & 0.160 & 0.011 & 0.002 & 0.002 \\ 0.060 & 0.127 & 0.135 & 0.189 & 0.343 & 0.094 & 0.012 & 0.004 \\ 0.029 & 0.060 & 0.075 & 0.110 & 0.190 & 0.335 & 0.138 & 0.029 \\ 0.014 & 0.028 & 0.032 & 0.064 & 0.099 & 0.270 & 0.355 & 0.144 \\ 0.008 & 0.016 & 0.019 & 0.040 & 0.078 & 0.153 & 0.315 & 0.456 \\ 0.005 & 0.010 & 0.012 & 0.022 & 0.048 & 0.092 & 0.153 & 0.348 \end{pmatrix}$$

$$
\boldsymbol{A_2} = \begin{pmatrix}
0.425 & 0.117 & 0.029 & 0.016 & 0.032 & 0.021 & 0.019 & 0.011 \\
0.269 & 0.308 & 0.233 & 0.062 & 0.021 & 0.014 & 0.003 & 0.001 \\
0.118 & 0.187 & 0.276 & 0.208 & 0.026 & 0.009 & 0.002 & 0.003 \\
0.075 & 0.138 & 0.177 & 0.276 & 0.151 & 0.011 & 0.002 & 0.002 \\
0.058 & 0.126 & 0.136 & 0.188 & 0.339 & 0.090 & 0.011 & 0.004 \\
0.029 & 0.063 & 0.080 & 0.116 & 0.197 & 0.330 & 0.131 & 0.027 \\
0.014 & 0.031 & 0.035 & 0.068 & 0.103 & 0.272 & 0.350 & 0.137 \\
0.008 & 0.019 & 0.020 & 0.043 & 0.081 & 0.157 & 0.322 & 0.451 \\
0.005 & 0.011 & 0.013 & 0.024 & 0.050 & 0.095 & 0.160 & 0.363
\end{pmatrix}
$$

$$
\boldsymbol{A_3} = \begin{pmatrix}
0.428 & 0.121 & 0.028 & 0.015 & 0.027 & 0.020 & 0.018 & 0.011 \\
0.266 & 0.309 & 0.238 & 0.064 & 0.017 & 0.011 & 0.002 & 0.001 \\
0.122 & 0.191 & 0.285 & 0.218 & 0.026 & 0.008 & 0.001 & 0.003 \\
0.077 & 0.140 & 0.177 & 0.278 & 0.159 & 0.010 & 0.001 & 0.002 \\
0.053 & 0.117 & 0.126 & 0.175 & 0.329 & 0.081 & 0.010 & 0.004 \\
0.029 & 0.062 & 0.078 & 0.115 & 0.204 & 0.328 & 0.122 & 0.026 \\
0.013 & 0.029 & 0.034 & 0.068 & 0.103 & 0.280 & 0.343 & 0.129 \\
0.008 & 0.018 & 0.020 & 0.043 & 0.083 & 0.164 & 0.335 & 0.447 \\
0.005 & 0.011 & 0.013 & 0.024 & 0.052 & 0.099 & 0.168 & 0.377
\end{pmatrix}
$$

$$
\boldsymbol{A_4} = \begin{pmatrix}
0.452 & 0.133 & 0.034 & 0.017 & 0.035 & 0.021 & 0.020 & 0.012 \\
0.253 & 0.301 & 0.243 & 0.067 & 0.020 & 0.012 & 0.003 & 0.001 \\
0.113 & 0.180 & 0.272 & 0.214 & 0.026 & 0.008 & 0.002 & 0.003 \\
0.071 & 0.133 & 0.169 & 0.266 & 0.144 & 0.011 & 0.002 & 0.002 \\
0.053 & 0.117 & 0.124 & 0.172 & 0.321 & 0.077 & 0.009 & 0.004 \\
0.030 & 0.067 & 0.084 & 0.120 & 0.207 & 0.319 & 0.113 & 0.025 \\
0.014 & 0.034 & 0.038 & 0.073 & 0.109 & 0.284 & 0.339 & 0.124 \\
0.008 & 0.021 & 0.022 & 0.046 & 0.086 & 0.167 & 0.340 & 0.441 \\
0.005 & 0.013 & 0.014 & 0.026 & 0.053 & 0.101 & 0.172 & 0.388
\end{pmatrix}
$$

In each matrix, the ***k***th column represents the fluorescence spectrum of ***Dyek*** (***k*** = ***1***, ***2***, ···, and ***8***) for each capillary. The nine elements in each column are normalized so that their summation is one. These four matrixes are equivalent, but they slightly different from each other. This is because the relative positions of each detection point and the corresponding lens are slightly different; therefore, incident angles of the collimated fluxes on each dichroic mirror are slightly different for the four capillaries. Since the eight columns of each of matrixes ***Aj*** are linearly independent, the following 8 × 9 general inverse matrixes $\boldsymbol{Aj}^{-}$ are derived by $(\boldsymbol{Aj}^{\mathrm{T}} \times \boldsymbol{Aj})^{-1} \times \boldsymbol{Aj}^{\mathrm{T}}$.

$$
\boldsymbol{A_1}^{-} = \begin{pmatrix}
3.334 & -1.873 & 1.921 & -1.382 & 0.430 & -0.166 & -0.102 & 0.011 & -0.078 \\
-3.719 & 8.413 & -9.777 & 7.457 & -3.376 & 0.890 & -0.020 & -0.308 & 0.503 \\
0.989 & -4.697 & 11.949 & -10.494 & 4.871 & -1.449 & 0.244 & 0.492 & -0.727 \\
0.128 & -0.197 & -3.693 & 8.637 & -4.354 & 1.270 & -0.244 & -0.415 & 0.559 \\
0.359 & -0.918 & 0.733 & -3.235 & 5.205 & -1.669 & 0.349 & 0.454 & -0.654 \\
-0.100 & 0.349 & -0.479 & 0.357 & -2.444 & 5.055 & -1.738 & -0.890 & 1.501 \\
0.031 & -0.151 & 0.466 & -0.596 & 1.141 & -3.708 & 4.705 & 0.704 & -2.580 \\
-0.022 & 0.075 & -0.122 & 0.259 & -0.487 & 0.824 & -2.277 & 1.115 & 2.293
\end{pmatrix}
$$

$$
\boldsymbol{A_2}^{-} = \begin{pmatrix}
3.243 & -2.008 & 2.237 & -1.687 & 0.538 & -0.178 & -0.104 & 0.044 & -0.107 \\
-3.547 & 8.715 & -11.019 & 8.761 & -3.775 & 0.892 & 0.057 & -0.459 & 0.631 \\
0.893 & -4.793 & 12.962 & -11.770 & 5.208 & -1.406 & 0.117 & 0.667 & -0.862 \\
0.125 & -0.228 & -3.824 & 9.098 & -4.371 & 1.166 & -0.133 & -0.511 & 0.615 \\
0.362 & -0.938 & 0.744 & -3.356 & 5.231 & -1.568 & 0.236 & 0.542 & -0.694 \\
-0.098 & 0.371 & -0.534 & 0.377 & -2.586 & 5.044 & -1.527 & -1.068 & 1.554 \\
0.044 & -0.197 & 0.570 & -0.701 & 1.247 & -3.758 & 4.521 & 0.997 & -2.674 \\
-0.023 & 0.080 & -0.139 & 0.282 & -0.508 & 0.854 & -2.252 & 0.940 & 2.376
\end{pmatrix}
$$

$$
\boldsymbol{A_3}^{-} = \begin{pmatrix}
3.226 & -2.082 & 2.330 & -1.864 & 0.723 & -0.199 & -0.109 & 0.068 & -0.133 \\
-3.396 & 8.583 & -10.755 & 8.911 & -4.194 & 0.878 & 0.135 & -0.539 & 0.672 \\
0.750 & -4.468 & 12.279 & -11.688 & 5.621 & -1.301 & -0.015 & 0.753 & -0.883 \\
0.150 & -0.420 & -3.414 & 8.877 & -4.614 & 1.057 & -0.020 & -0.573 & 0.631 \\
0.343 & -0.867 & 0.666 & -3.189 & 5.340 & -1.393 & 0.091 & 0.612 & -0.707 \\
-0.094 & 0.356 & -0.475 & 0.341 & -2.732 & 4.897 & -1.219 & -1.238 & 1.578 \\
0.039 & -0.172 & 0.501 & -0.679 & 1.426 & -3.824 & 4.296 & 1.331 & -2.798 \\
-0.019 & 0.071 & -0.134 & 0.293 & -0.589 & 0.936 & -2.247 & 0.733 & 2.492
\end{pmatrix}
$$

$$
\boldsymbol{A_4}^{-} = \begin{pmatrix}
3.074 & -2.272 & 2.747 & -2.200 & 0.730 & -0.160 & -0.157 & 0.112 & -0.169 \\
-3.195 & 9.185 & -12.590 & 10.591 & -4.509 & 0.759 & 0.344 & -0.804 & 0.895 \\
0.684 & -4.704 & 13.680 & -13.094 & 5.717 & -1.122 & -0.265 & 1.032 & -1.099 \\
0.165 & -0.446 & -3.654 & 9.280 & -4.403 & 0.876 & 0.135 & -0.699 & 0.709 \\
0.326 & -1.016 & 0.950 & -3.497 & 5.427 & -1.290 & -0.061 & 0.740 & -0.783 \\
-0.090 & 0.412 & -0.651 & 0.429 & -2.898 & 4.907 & -0.945 & -1.436 & 1.643 \\
0.054 & -0.241 & 0.692 & -0.826 & 1.515 & -3.889 & 4.042 & 1.624 & -2.899 \\
-0.018 & 0.085 & -0.178 & 0.323 & -0.601 & 0.971 & -2.181 & 0.568 & 2.572
\end{pmatrix}
$$

For ***Capj***, when fluorescence intensities of ***Dye1***−***Dye8*** and ***C1***−***C9*** are denoted by I***j***(***Dye1***)−I***j***(***Dye8***) and I***j***(***C1***)−I***j***(***C9***) at each time point, respectively, the following 8 × 1-dimensional vector ***Xj*** and 9 × 1-dimensional vector ***Yj*** are defined as

$$
\boldsymbol{X_j} = \begin{pmatrix}
\mathrm{I}_j(\boldsymbol{Dye1}) \\
\mathrm{I}_j(\boldsymbol{Dye2}) \\
\vdots \\
\mathrm{I}_j(\boldsymbol{Dye8})
\end{pmatrix}
$$

$$Y_j = \begin{pmatrix} I_j(C1) \\ I_j(C2) \\ \vdots \\ I_j(C9) \end{pmatrix}$$

The following equations thus hold at each time point:

$$Y_j = A_j \times X_j \qquad \text{(S1)}$$

$$X_j = A_j^{-} \times Y_j \qquad \text{(S2)}$$

The time courses of Ij($C1$)−Ij($C9$), i.e., the nine-color electropherograms in Fig. 7a, were converted to the time courses of Ij($Dye1$)−Ij($Dye8$), i.e., the eight-dye electropherograms in Fig. 7c, by Equation (S2).

Emission spectra of light sources

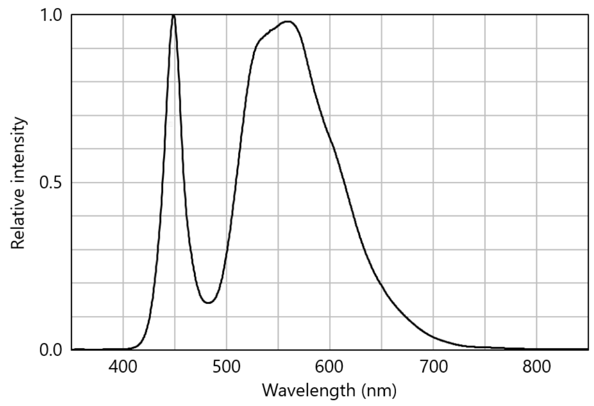

Fig. S3: Spectrum of LED white light

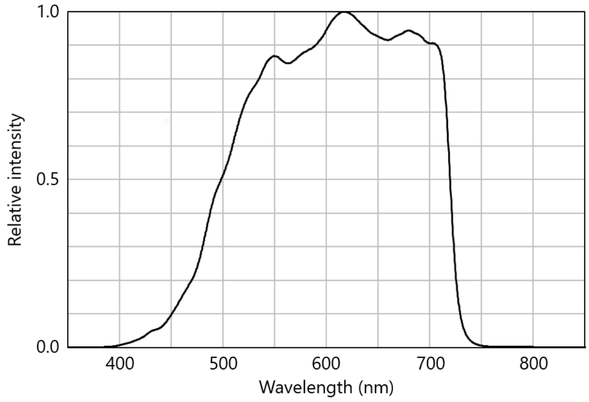

Fig. S4: Spectrum of halogen-lamp light transmitted through the shortpass filter with cut-off wavelength of 700 nm
